# Changes in body mass index among pregnant women during labor over four decades: a retrospective longitudinal analysis

**DOI:** 10.1038/s41366-026-02021-6

**Published:** 2026-02-04

**Authors:** Petra Gašparová, Zuzana Ballová, Martina Sitáš, Erik Dosedla

**Affiliations:** Department of Gynecology and Obstetrics, AGEL Košice-Šaca University Hospital, Faculty of Medicine, Pavol Jozef Šafárik University in Košice, Košice, Slovakia

**Keywords:** Risk factors, Health care

## Abstract

**Introduction:**

The global rise in maternal obesity presents a growing public health challenge, with significant implications for pregnancy outcomes. This study offers a comprehensive retrospective analysis of changes in Body Mass Index (BMI) among pregnant women during labor over four decades.

**Material and methods:**

BMI data were retrospectively collected from 13,193 pregnant women divided into three cohorts: Group 0 (1986–1990), Group 1 (2009–2013), and Group 2 (2024).

**Results:**

Mean BMI increased significantly from 23.75 kg/m² in Group 0 to 27.33 kg/m² in Group 2 (*p* < 0.001). The prevalence of obesity rose more than fivefold, from 4.87% to 25.76%. Regression analysis confirmed a linear and statistically significant upward trajectory across all groups.

**Conclusions:**

The sustained increase in maternal BMI underscores the urgency of implementing targeted interventions, including preconception counseling and personalized antenatal care, to mitigate the adverse maternal and neonatal outcomes associated with elevated BMI.

## Introduction

Body Mass Index (BMI) has long been recognized as a key indicator of nutritional status and overall health [1]. Obesity is a global health challenge, with its prevalence steadily increasing among pregnant women, particularly in Western countries [2]. Obesity is defined as a BMI of ≥30.0 kg/m² and is further classified into three categories: class I (BMI 30.0–34.9 kg/m²), class II (BMI 35.0–39.9 kg/m²), and class III, or severe obesity, with a BMI of ≥40.0 kg/m² [3].

In obstetrics, BMI during pregnancy is particularly significant, as it influences both maternal and neonatal outcomes, including risks associated with labor and delivery [4]. Over the past three decades, a global trend toward higher BMI has been observed, reflecting shifts in lifestyle, dietary patterns, and socioeconomic factors [5].

Globally, the prevalence of overweight and obesity has risen steadily. Between 1980 and 2008, the global mean BMI increased by ~0.4 kg/m² per decade for men and 0.5 kg/m² for women [6]. By 2022, more than half of adults in the European Union were classified as overweight [7]. This trend has also affected pregnant women, who face unique challenges related to weight gain during pregnancy and its management during labor. Obesity in pregnancy is associated with adverse outcomes such as gestational diabetes, hypertension, preeclampsia, and an increased likelihood of instrumental delivery, cesarean section, and stillbirth [2, 8]. The rising prevalence of high BMI in pregnant women is not merely a health issue but also an economic and societal challenge.

In Slovakia, similar trends have been observed. A 2021 survey indicated that 41% of the population had a BMI of 27 kg/m² or higher, with 23% of adults classified as having obesity (BMI ≥ 30.0 kg/m²) [9]. For pregnant women, the implications of these trends are profound, as BMI directly influences maternal health, labor dynamics, and neonatal outcomes. Despite increasing awareness and healthcare interventions, the upward trajectory of BMI remains a critical concern.

This study uses data spanning four decades to analyze changes in BMI among pregnant women during labor. The data is categorized into three groups: Group 0 (1986–1990), Group 1 (2009–2013), and Group 2 (2024). These groups provide a timeline to examine shifts in mean BMI, the distribution of BMI categories, and their statistical significance. This longitudinal approach offers a comprehensive understanding of how BMI trends have evolved and the underlying factors contributing to these changes.

This analysis aims to answer the critical question: How has BMI changed among pregnant women during labor over the past four decades? The study focuses on these changes, with broader implications for maternal and neonatal health, including the impact of high BMI on the mode of delivery, discussed in the context of the results.

## Material and methods

### Data collection

The study analyzed BMI data from 13,193 pregnant women during labor over a 30-year period, categorized into three-time frames. Data for 1986–1990 were retrieved from handwritten delivery logbooks and archived patient charts at the Department of Obstetrics and Gynecology, AGEL University Hospital Košice-Šaca, Slovakia. Data for 2009–2013 and 2024 were extracted from the hospital’s electronic medical record system. All data were collected from the same institution to ensure consistency. These periods correspond to major institutional and policy changes; continuous annual data were unavailable, but a regression trend is provided (Table 1):

**Table 1:** Maternal demographic and clinical characteristics by study group.

Group	Maternal age (years, mean ± SD)	GA at delivery (weeks, mean ± SD)	Primiparous (%)	Multiparous (%)
1986–1990	24.19 ± 5.35	38.81 ± 2.29	35.8	64.2
2009–2013	27.84 ± 7.02	38.75 ± 2.15	44.1	55.9
2024	30.24 ± 7.15	38.68 ± 2.27	43.7	56.3

Maternal age and gestational age (GA) at delivery are presented as mean ± standard deviation (SD). Values for primiparous and multiparous women are expressed as percentages. All pregnancies included in the analysis were singleton gestations.

Group 0 (4 864) (1986–1990): Data from this group represent an earlier period when mean BMI values were relatively low.

Group 1 (7 330 patients) (2009–2013): This group reflects transitional BMI trends.

Group 2 (1 000) (2024): Recent data illustrate current BMI trends.

### Statistical analysis

Descriptive Statistics (means, standard deviations, and BMI category distributions: normal weight, overweight, obesity) were calculated for each group. BMI was assessed upon admission to the labor ward before delivery.

Comparisons between groups were conducted using one-way ANOVA. Linear regression analysis was applied to evaluate temporal trends. Statistical significance was set at *p* < 0.001. All analyses were performed using IBM SPSS Statistics, version 29.0.

## Results

**Fig. 1:**
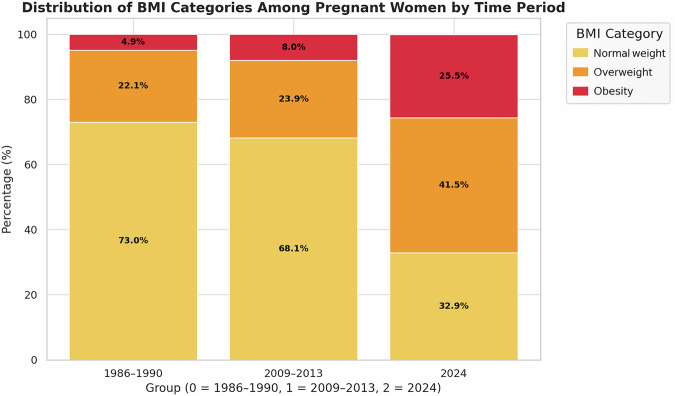
Stacked bar chart showing the distribution of BMI categories (Normal weight, Overweight, and Obesity) among pregnant women across the three time periods (1986–1990, 2009–2013, and 2024).

A total of 13,193 pregnant women were included in the analysis, distributed across three temporal cohorts: Group 0 (1986–1990), Group 1 (2009–2013), and Group 2 (2024). The study revealed a significant increase in the BMI of pregnant women during labor over the past four decades (Fig. 1). According to Table 2, the mean BMI rose from 23.75 in Group 0 to 24.21 in Group 1, reaching 27.33 in Group 2. All pairwise comparisons revealed statistically significant differences (*p* < 0.001), indicating a robust upward trend in maternal BMI over the last four decades. The proportion of women with normal weight (18.5–24.9 kg/m²) decreased significantly over time. In Group 0, 73.04% of women fell into the normal weight category, compared to 68.13% in Group 1 and only 32.32% in Group 2. The reductions between Group 0 and Group 1, and between Group 1 and Group 2, were both statistically significant (*p* < 0.001), reflecting a marked shift in population distribution toward higher BMI categories. The prevalence of overweight women (BMI 25.0–29.9 kg/m²) increased from 22.09% in Group 0 to 23.90% in Group 1 and further to 41.92% in Group 2. These increases were statistically significant both between Group 0 and Group 1 (*p* = 0.002) and between Group 1 and Group 2 (*p* < 0.001). Notably, the proportion of women with obesity (BMI ≥ 30.0 kg/m²) rose sharply over the study period. In Group 0, 4.87% of women were classified as having obesity, increasing to 7.97% in Group 1 and to 25.76% in Group 2. All pairwise comparisons were statistically significant (*p* < 0.001), indicating a more than fivefold increase in maternal obesity rates over the analyzed period.

**Table 2:** Distribution of BMI categories among pregnant women across three time periods (1986–1990, 2009–2013, 2024), including mean BMI and associated *p*-values.

Group	Mean BMI (*p*-value)	Normal weight % (*p*-value)	Overweight % (*p*-value)	Obesity % (*p*-value)
1986–1990	23.75	73.04%	22.09%	4.87%
2009–2013	24.21 (<0.001)	68.13% (<0.001)	23.90% (0.002)	7.97% (<0.001)
2024	27.33 (<0.001)	32.32% (<0.001)	41.92% (<0.001)	25.76% (<0.001)

**Fig. 2:**
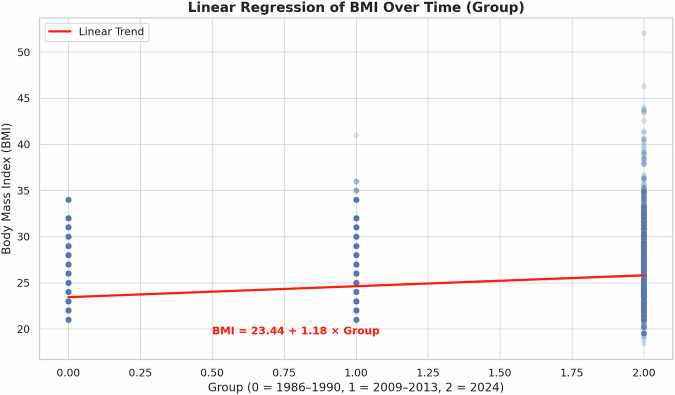
Scatter plot of individual BMI values across three temporal cohorts with linear regression trend line. Each point represents the BMI of a pregnant woman at the time of labor, grouped by cohort (1986–1990, 2009–2013, and 2024). The red line represents the linear regression fit, indicating a statistically significant upward trend in BMI over time (*p* < 0.001). The regression model demonstrates an estimated increase of 1.18 kg/m² per cohort, confirming a progressive rise in maternal BMI over the past four decades.

Figure 2 presents a scatter plot of individual BMI values across the three temporal cohorts—1986–1990, 2009–2013, and 2024—accompanied by a linear regression trend line. The data demonstrate a clear and statistically significant upward trajectory in maternal BMI over time.

The red regression line indicates a positive linear association between cohort year and BMI, with a slope consistent with prior parametric analyses. Specifically, the model estimates an increase of ~1.18 kg/m² per time group, reflecting a progressive elevation in maternal weight over the study period. The linear fit remained significant (*p* < 0.001), further validating the trend observed in descriptive and inferential statistics.

Notably, the dispersion of individual data points illustrates a broadening spread in BMI values in the most recent cohort (2024), suggesting not only a rise in average BMI but also increasing variability. This widening distribution may reflect growing disparities in maternal weight profiles and warrants further investigation into associated sociodemographic or behavioral factors.

Overall, the linear regression visualization confirms a systematic and accelerating rise in maternal BMI across decades, reinforcing concerns about the escalating prevalence of overweight and obesity in the obstetric population.

## Discussion

The data indicate a statistically significant and clinically relevant increase in BMI among pregnant women over the past four decades, driven by rising rates of overweight and obesity. The sharp drop in normal-weight pregnancies highlights the need for public health interventions focused on preconceptional and antenatal weight management.

Key contributors to this trend include changes in dietary habits, decreased physical activity, and shifts in socioeconomic conditions [10]. Urbanization and the widespread adoption of sedentary lifestyles have played a significant role in weight gain, especially among women of reproductive age. Furthermore, the increasing reliance on processed and calorie-dense foods has only intensified the problem [11].

The increase in the prevalence of high BMI among pregnant women poses significant public health concerns. Overweight and obesity affect approximately two-thirds of pregnancies, raising the risk of hypertension, preeclampsia, gestational diabetes, venous thromboembolism, and both maternal and fetal mortality [8, 12]. Moreover, maternal obesity is linked to an increased risk of future cardiovascular diseases for the mother [13].

Elevated BMI in pregnant women is closely linked to complications during labor and delivery. Maternal obesity increases the risk of adverse perinatal outcomes, including a lower probability of spontaneous labor onset, a higher rate of labor induction, and a higher likelihood of cesarean delivery, often due to labor dystocia and fetal macrosomia. It is also associated with an increased risk of stillbirth, a greater incidence of fetal macrosomia, and a higher frequency of urgent cesarean sections [13]. High BMI is associated with an increased risk of operative vaginal delivery and shoulder dystocia [14]. The risk of cesarean sections is 50% higher in women with overweight and more than twice as high in women with obesity compared to those with a normal weight [15].

Newborns of women with obesity have a higher risk of preterm birth, leading to lower gestational age. On the other hand, they are more likely to be large, which raises the risk of birth trauma. Additionally, they have a higher likelihood of low Apgar scores and more commonly experience hypoglycemia. The chances of admission to the neonatal intensive care unit or the need for specialized care are also greater [16]. Maternal obesity is further linked to an increased risk of birth defects in offspring, including heart defects, neural tube defects, and other congenital malformations. Moreover, children born to mothers with obesity are at higher risk of developing obesity, coronary heart disease, stroke, type 2 diabetes, and asthma. There is also evidence suggesting that maternal obesity may contribute to poorer cognitive development and a greater likelihood of neurodevelopmental disorders, such as cerebral palsy [17]. These children also have a higher risk of stillbirth and neonatal mortality. Perinatal mortality, which includes the death of the fetus or newborn, is almost six times higher in women with severe obesity (>40.0 kg/m²) compared to women with a BMI of 30.0–34.9 kg/m² [16].

Furthermore, the findings of our study are consistent with the projected epidemiological trends reported by Eurostat, which forecast a continued rise in maternal obesity in Slovakia through 2035. Notably, the rate of increase in Slovakia has outpaced the European average over the past decade, underscoring an urgent national public health priority [7]. These outcomes not only pose risks to maternal and neonatal health but also place a significant strain on healthcare systems. Addressing this requires a multi-pronged approach: preconception counseling and weight management programs can help women achieve a healthy BMI before pregnancy, while healthcare providers should offer personalized nutrition and exercise guidance to manage weight gain during pregnancy. Furthermore, public health campaigns must focus on raising awareness about the risks of obesity in pregnancy and the importance of maintaining a healthy lifestyle [18].

## Conclusion

The Institute of Medicine (IOM) recommends that women with a normal pre-pregnancy BMI (18.5–24.9) should gain between 11.5 and 16 kg during pregnancy. For women with overweight (BMI 25–29.9), the recommended weight gain is 7–11.5 kg, and for those with obesity (BMI ≥ 30.0), the recommended gain is 5–9 kg. These guidelines aim to help manage weight gain during pregnancy and reduce the risks associated with excessive weight gain [19].

The significant increase in BMI among pregnant women during labor over the past four decades highlights a pressing public health concern. This trend underscores the need for targeted interventions to address the underlying causes of rising BMI and mitigate its impact on maternal and neonatal health. By prioritizing education, prevention, and tailored care, healthcare systems can better support pregnant women and improve outcomes for future generations.

## Data Availability

The datasets generated and analyzed during the current study are available from the corresponding author upon reasonable request. All data are shared for non-commercial purposes in accordance with participant confidentiality and ethical regulations.
